# The bidirectional relationship between physical exercise and social self-efficacy: a cross-lagged model analysis of the mediating role of peer acceptance

**DOI:** 10.3389/fpsyg.2025.1701902

**Published:** 2025-11-19

**Authors:** Zexi Zhao, Ruoyu Li, Zi Ying, Aonan Yang

**Affiliations:** 1 The Department of Physical Education, Zhejiang University of Finance and Economics, Hangzhou, China; 2 Department of Physical Education, Jinzhong College of Information, Jinzhong, China; 3 School of Teacher Education (Physical Education), Taizhou University, Taizhou, China; 4 School of Physical Education, Shanghai University of Sport, Shanghai, China

**Keywords:** physical exercise, social self-efficacy, peer acceptance, longitudinal mediation, college students

## Abstract

**Objective:**

Social self-efficacy is a crucial indicator of college students’ mental health, yet the underlying mechanisms of how physical exercise influences it require deeper exploration. This study aimed to examine the relationship between physical exercise and social self-efficacy among college students through a longitudinal design and test the mediating role of peer acceptance.

**Methods:**

A three-wave longitudinal tracking design was employed with 758 undergraduates from Zhengzhou University, with surveys conducted at 3-month intervals. Data were collected using the Physical Activity Rating Scale, Social Self-Efficacy Scale, and Peer Acceptance Scale. Cross-lagged models and bootstrap methods were used for statistical analysis.

**Results:**

① Significant bidirectional longitudinal predictive relationships existed between physical exercise and social self-efficacy (*β* = 0.19–0.24, *p* < 0.01; *β* = 0.13–0.16, *p* < 0.01); ② Peer acceptance played a significant longitudinal mediating role in their relationship, with T1 physical exercise predicting T3 social self-efficacy through T2 peer acceptance [*β* = 0.071, 95% CI = (0.038,0.108)], and T1 social self-efficacy predicting T3 physical exercise through T2 peer acceptance [*β* = 0.053, 95% CI = (0.025,0.084)].

**Conclusion:**

Physical exercise and social self-efficacy form a mutually reinforcing dynamic cycle, with peer acceptance serving as a key mediating mechanism. This study provides scientific evidence for enhancing college students’ social adaptation through creating positive peer environments.

## 1 Introduction

The United Nations Sustainable Development Goals (SDGs) highlight “Good Health and Well-Being” as a core element of universal health coverage, emphasizing the critical importance of mental health (Heymann and Sprague, 2023). In China, where higher education has been rapidly expanding, the large-scale population of university students means that their mental health is not only central to individual growth and well-being but also crucial to the quality of future human capital and the nation’s long-term social development (Zhang and Zhao, 2024). University students often face multiple challenges, including academic competition, employment pressure, and changes in interpersonal relationships. Consequently, researchers and practitioners have increasingly focused on their capacity for psychological adaptation, particularly their level of social self-efficacy (Wang et al., 2024).

Social self-efficacy refers to an individual’s belief in their ability to initiate and maintain the behaviors necessary to achieve desired goals in social interactions (Tak et al., 2017). It is a core psychological resource for adapting to complex social environments, building positive relationships, and achieving personal development goals. Previous research has shown significant individual differences in the social self-efficacy of Chinese college students. A considerable proportion exhibit tendencies toward social withdrawal or avoidance, difficulties in interpersonal communication, and maladaptation to new environments (He and Wang, 2025). Enhancing social self-efficacy is therefore essential for helping students integrate into society, build healthy relationships, and succeed in their careers (Tung and Huong, 2023). Students with high social self-efficacy are more likely to receive social support, adapt successfully to university life, and experience lower levels of depression (Meng et al., 2015). Furthermore, social self-efficacy is positively associated with career competence and subjective career success, giving socially confident students a competitive advantage in the job market (Anderson and Betz, 2001).

Physical exercise, as a structured activity rich in social interaction, has been widely recognized for its benefits to psychosocial development (Ahmed et al., 2021; Li and Huang, 2025). From the perspective of Social Cognitive Theory, the physical activity environment serves as an effective platform for social learning. In team sports, individuals frequently encounter and address various social tasks, such as cooperation, conflict resolution, adherence to rules, and communication (Bean et al., 2022). Each successful experience, whether achieving a team goal or resolving a disagreement, can be translated into a positive self-assessment of one’s social abilities, thereby directly enhancing social self-efficacy (Tak et al., 2017). Moreover, regular physical activity can improve mood, reduce anxiety and depression, and foster emotional stability, which in turn provides a psychological foundation for building social confidence and engaging in social initiatives (Xing and Ge, 2025).

Importantly, the enhancement of social self-efficacy through physical activity is not solely dependent on individual experiential gains. Peer acceptance plays a crucial mediating role in this process. Peer acceptance refers to the degree to which an individual is recognized, liked, and welcomed within their peer group (Douma et al., 2025). In physical activity contexts, especially in team-based sports, participants engage in frequent interactions through joint training, collaborative competition, and shared experiences. These interactions provide opportunities to showcase and refine social skills, making it easier to gain positive evaluations and emotional support from peers (Jones, 2024; Zhou et al., 2024). Positive feedback and acceptance from peers reinforce individuals’ beliefs in their social capabilities, thereby fostering higher levels of social self-efficacy. When individuals feel highly accepted within a sports team, a positive cycle can form: acceptance enhances confidence and willingness to engage socially, leading to more frequent and successful interactions, which in turn further consolidate peer status and acceptance, ultimately enhancing social self-efficacy (Morgan and Parker, 2017). Conversely, experiences of rejection or indifference can undermine social confidence, creating a downward spiral of reduced self-efficacy (Erol and Orth, 2016; Harris and Orth, 2020).

However, existing research has notable limitations. First, much of the literature on the relationships among physical exercise, peer acceptance, and social self-efficacy relies on cross-sectional designs, making it difficult to establish causal and temporal dynamics between variables (Xu et al., 2024; Zhou et al., 2025). As an intervention requiring sustained engagement, the benefits of physical activity, particularly those related to peer relationships and social self-efficacy, are likely to accumulate and emerge over time. Single-time-point data cannot capture the full scope of these effects or the developmental trajectory of the underlying mechanisms (Du et al., 2025). Second, there remains a lack of empirical validation for the longitudinal mediation pathway “Physical Exercise → Peer Acceptance → Social Self-Efficacy” (Xing and Ge, 2025). As an important contextual variable linking external behavioral engagement (physical activity) to internal psychological states (social self-efficacy), the dynamic mediating effect of peer acceptance warrants rigorous longitudinal testing.

To address these gaps and elucidate the underlying mechanisms, this study employed a three-wave longitudinal design (6-month intervals) focusing on a sample of university students. Using a cross-lagged panel model, we aimed to investigate the following questions: (1) Can baseline levels and changes in physical activity predict subsequent changes in peer acceptance and social self-efficacy? (2) Does peer acceptance serve as a significant longitudinal mediator in the relationship between physical exercise and social self-efficacy? (3) Are there dynamic, time-dependent reciprocal effects among the variables? For example, does higher early social self-efficacy lead to greater subsequent participation in physical activity?

Theoretically, this study makes three key contributions: First, it fills an empirical gap by providing longitudinal evidence for the pathway “Physical Exercise → Peer Acceptance → Social Self-Efficacy,” moving beyond the limitations of cross-sectional designs. Second, it extends Social Cognitive Theory’s application in sport psychology by introducing peer acceptance as a critical contextual mediator linking behavioral engagement to psychological outcomes. Third, it reveals a bidirectional reinforcement mechanism between physical exercise and social self-efficacy, offering a more nuanced theoretical perspective than previous unidirectional causal models. Practically, the findings offer valuable insights for university mental health educators, sports departments, and student affairs administrators: by intentionally designing sports programs (especially team-based activities) that emphasize cooperation, communication, and role-taking, and by fostering a positive, inclusive, and supportive peer environment, it is possible to leverage peer acceptance as a key mechanism to enhance students’ social self-efficacy, thereby improving their overall psychosocial adaptation and promoting healthy development.

## 2 Participants and methods

### 2.1 Participants

A cluster-randomized sampling method was employed to select undergraduates from Zhengzhou University as participants. Stratified sampling was used to ensure a balanced distribution across academic years, covering first- through fourth-year students. The three waves of longitudinal data collection took place in June 2024 (T1), September 2024 (T2), and December 2024 (T3), with a 3-month interval between each wave. A total of 847, 823, and 839 valid questionnaires were collected in T1, T2, and T3, respectively. The 3-month measurement interval corresponds with the standard 2–4-month period necessary for behavioral changes to stabilize, as indicated by the Transtheoretical Model. It is supported by prior empirical studies in similar contexts. This duration balances the need to detect meaningful change while minimizing memory effects, participant attrition, and logistical challenges in an academic setting.

Invalid responses were excluded based on the following criteria: (1) more than two-thirds of items unanswered; (2) failure to pass logical consistency checks; (3) patterned responding (e.g., selecting the same option for all items); and (4) incomplete demographic information. Ultimately, 758 participants completed all three surveys with matched data, yielding an attrition rate of 10.51%, primarily due to internships, leaves of absence, or transfers.

Independent-samples t-tests showed no significant differences between participants lost to follow-up and those retained in T1 scores of physical exercise (*t* = −0.127, *p* > 0.05), social self-efficacy (*t* = 0.853, *p* > 0.05), peer acceptance (*t* = −0.943, *p* > 0.05), and study engagement (*t* = −1.324, *p* > 0.05), indicating no systematic attrition bias. The study was approved by the institutional ethics committee, with informed consent obtained from students, supervisors, and the university. Demographic characteristics are presented in Table 1.

**Table 1:** Demographic characteristics of participants (*N* = 847).

Variable	Category	*n*	%
Gender	Male	371	48.9
	Female	387	51.1
Year of study	First-year	203	26.8
	Second-year	196	25.9
	Third-year	181	23.9
	Fourth-year	178	23.5
Major	Science & Engineering	312	41.2
	Humanities	189	24.9
	Medicine	142	18.7
	Others	115	15.2
Place of origin	Urban	342	45.1
	Rural	416	54.9

### 2.2 Methods

#### 2.2.1 Physical Activity Rating Scale

This study employed the widely used Physical Activity Rating Scale-3 (PARS-3) (Ren et al., 2021; Yang et al., 2021), which assesses the amount of physical exercise from three dimensions: intensity, duration, and frequency. Furthermore, items adapted from other physical activity questionnaires were incorporated to measure four further aspects: attitude toward exercise, exercise-related cognition, initiative in participation, and post-exercise experience. Responses were rated on a 5-point Likert scale (1 = “strongly disagree” to 5 = “strongly agree”), with higher scores indicating higher levels of physical activity. In the present study, the internal consistency coefficients of the scale across the three measurement waves were 0.846 (T1), 0.821 (T2), and 0.793 (T3).

#### 2.2.2 Personal Social Self-Efficacy Scale

Personal social self-efficacy was assessed using the Personal Social Self-Efficacy Scale (PSSE) developed by Smith and Betz (2000). The scale comprises 18 items with a single-factor structure and factor loadings ranging from 0.52 to 0.78. A 5-point Likert scale (1 = “strongly disagree” to 5 = “strongly agree”) was used, indicating that elevated scores correspond to higher levels of personal social self-efficacy. In this study, the Cronbach’s *α* coefficients for the three measurement waves were 0.934 (T1), 0.941 (T2), and 0.928 (T3).

#### 2.2.3 Peer Acceptance Scale

Peer acceptance was measured using the revised Peer Relations Scale (Kistner et al., 1999; Oberle et al., 2010), which includes two subscales—peer acceptance and peer fear/inferiority—totaling 30 items. In this study, only items 1–20 (the peer acceptance subscale) were used. Items were rated on a 4-point scale (1 = “strongly disagree” to 4 = “strongly agree”), with all items reverse-scored except for items 1, 3, 7, 11, and 17. Higher total scores indicate greater peer acceptance, better peer relationships, and higher popularity within the group. The Cronbach’s α coefficients for the peer acceptance subscale across the three measurement waves were 0.891 (T1), 0.903 (T2), and 0.897 (T3).

### 2.3 Procedure

Before each wave of data collection, the researchers explained the purpose and procedures of the study to the participants and obtained their informed consent. Participants were then provided with the questionnaires and instructed to read the guidelines carefully before responding based on their actual situation. Upon completion, the researchers collected questionnaires on the spot. All measurements were conducted in the same classroom environment to ensure consistent testing conditions across waves.

### 2.4 Data analysis

All analyses were conducted using SPSS 26.0 and Mplus 8.3. After assessing common method bias via Harman’s single-factor test, descriptive statistics and Pearson correlations were computed to examine interrelationships among the three-wave variables. A cross-lagged panel model was then applied to investigate the bidirectional relationships between physical exercise and social self-efficacy, as well as the mediating role of peer acceptance.

The cross-lagged model assesses how earlier measures of one variable predict later measures of another while controlling for autoregressive effects and covariates, thereby providing stronger causal inference than cross-sectional designs.

Preliminary correlation analyses showed that, among the demographic variables collected, only place of origin (rural/urban) was modestly correlated with the key study variables. Therefore, in line with methodological recommendations that control variables should be selectively included based on substantive correlation with outcomes, we incorporated place of origin as a time-invariant covariate influencing all T1 variables. Other demographic factors (gender, year of study, and major) were excluded due to negligible associations.

Model fit was evaluated using multiple indices: *χ*^2^/df < 3, CFI & TLI ≥ 0.90, RMSEA < 0.08, and SRMR < 0.08. Mediation effects were tested using bootstrapping with 5,000 resamples; 95% CIs excluding zero indicate statistical significance.

## 3 Results

### 3.1 Common method bias test

To reduce the potential influence of common method bias, both procedural and statistical control measures were implemented. This study stressed anonymity and confidentiality in its procedure and used reverse-scored items in the questionnaires. Statistically, Harman’s single-factor test was conducted using all items from the three scales. The results indicated that 16 factors had eigenvalues greater than 1, and the variance explained by the first factor was 27.46%, which is below the critical threshold of 40%. These findings indicate that common method bias was not a major issue in this study.

### 3.2 Descriptive statistics and correlation analysis

Table 2 presents the means, standard deviations, and Pearson correlation coefficients of physical exercise, social self-efficacy, and peer acceptance at all three measurement points. The results revealed that physical exercise at T1, T2, and T3 was significantly positively correlated with social self-efficacy and peer acceptance at the same and subsequent time points (*p* < 0.01). Similarly, social self-efficacy was significantly positively correlated with peer acceptance across the three waves (*p* < 0.01).

**Table 2:** Means, standard deviations, and correlations among main variables (*N* = 758).

Variable	M ± SD	1	2	3	4	5	6	7	8	9
Physical Exercise T1	3.34 ± 0.78	1								
Social Self-Efficacy T1	3.12 ± 0.58	0.58**	1							
Peer Acceptance T1	3.45 ± 0.71	0.52**	0.48**	1						
Physical Exercise T2	3.26 ± 0.73	0.61**	0.39**	0.43**	1					
Social Self-Efficacy T2	3.08 ± 0.52	0.41**	0.56**	0.47**	0.57**	1				
Peer Acceptance T2	3.51 ± 0.69	0.47**	0.44**	0.63**	0.54**	0.59**	1			
Physical Exercise T3	3.29 ± 0.75	0.38**	0.31**	0.35**	0.49**	0.43**	0.41**	1		
Social Self-Efficacy T3	3.11 ± 0.54	0.33**	0.29**	0.34**	0.42**	0.41**	0.44**	0.51**	1	
Peer Acceptance T3	3.56 ± 0.67	0.39**	0.28**	0.36**	0.51**	0.47**	0.52**	0.61**	0.62**	1

M, Mean; SD, Standard Deviation; T1–T3, Time 1–Time 3. **p < 0.01.

### 3.3 Cross-lagged model analysis

A longitudinal measurement invariance test was conducted to examine the cross-time stability of the Physical Exercise Scale, Social Self-Efficacy Scale, and Peer Acceptance Scale. Following the criteria proposed by Cheung et al. (ΔCFI ≤ 0.01 and ΔRMSEA ≤ 0.015) and adopting the item parceling strategy recommended by Wu et al. to simplify the model, we sequentially tested configural invariance (M1), weak invariance (M2), strong invariance (M3), and strict invariance (M4).

The results (Table 3) indicate that changes in fit indices across all models met the established criteria (ΔCFI < 0.01, ΔRMSEA < 0.015), suggesting that the three scales achieved longitudinal measurement invariance across the three measurement waves. This finding supports the validity of conducting subsequent cross-lagged analyses.

**Table 3:** Results of longitudinal measurement invariance testing.

Variable	Model	*χ* ^2^	df	CFI	TLI	RMSEA (90% CI)	SRMR	MC	ΔCFI	ΔRMSEA
Physical Exercise	M1: Configural invariance	198.543	132	0.982	0.974	0.026[0.019,0.032]	0.031			
	M2: Weak invariance	213.867	140	0.981	0.975	0.027[0.020,0.033]	0.044	M1vs. M2	0.01	0.01
	M3: Strong invariance	236.492	146	0.977	0.971	0.029[0.023,0.035]	0.045	M2vs. M3	0.004	0.002
	M4: Strict invariance	267.334	154	0.971	0.965	0.032[0.026,0.037]	0.047	M3vs. M4	0.006	0.003
Social Self-Efficacy	M1: Configural invariance	156.789	87	0.943	0.921	0.033[0.026,0.040]	0.038			
	M2: Weak invariance	172.634	93	0.938	0.919	0.034[0.027,0.041]	0.047	M1vs. M2	0.005	0.01
	M3: Strong invariance	189.456	97	0.932	0.915	0.035[0.029,0.042]	0.049	M2vs. M3	0.006	0.01
	M4: Strict invariance	208.723	103	0.926	0.912	0.037[0.030,0.043]	0.052	M3vs. M4	0.006	0.002
Peer Acceptance	M1: Configural invariance	543.892	267	0.951	0.936	0.037[0.032,0.041]	0.043			
	M2: Weak invariance	571.234	275	0.947	0.933	0.038[0.033,0.043]	0.054	M1vs. M2	0.004	0.01
	M3: Strong invariance	598.567	281	0.943	0.930	0.039[0.034,0.044]	0.055	M2vs. M3	0.004	0.01
	M4: Strict invariance	627.843	289	0.938	0.927	0.040[0.035,0.045]	0.056	M3vs. M4	0.005	0.01

### 3.4 Cross-lagged model construction and testing between physical exercise and social self-efficacy

To explore the directional relationship between physical exercise and social self-efficacy, a cross-lagged panel model was constructed based on the correlation analysis. The model demonstrated a good fit to the data, *χ*^2^ = 6.34, df = 4, *χ*^2^/df = 1.59, *p* < 0.01, CFI = 0.99, GFI = 0.99, SRMR = 0.02, RMSEA = 0.03.

The autoregressive effects indicated that physical exercise at a prior measurement (Tn) significantly predicted physical exercise at the subsequent measurement (Tn + 1), and that social self-efficacy at a Tn significantly predicted social self-efficacy at the subsequent measurement. Specifically, T1 physical exercise significantly and positively predicted T2 physical exercise (*β* = 0.57, *p* < 0.01), and T2 physical exercise significantly predicted T3 physical exercise (*β* = 0.42, *p* < 0.01). Similarly, T1 social self-efficacy significantly predicted T2 social self-efficacy (*β* = 0.51, *p* < 0.01), and T2 social self-efficacy significantly predicted T3 social self-efficacy (*β* = 0.38, *p* < 0.01).

**Figure 1:**
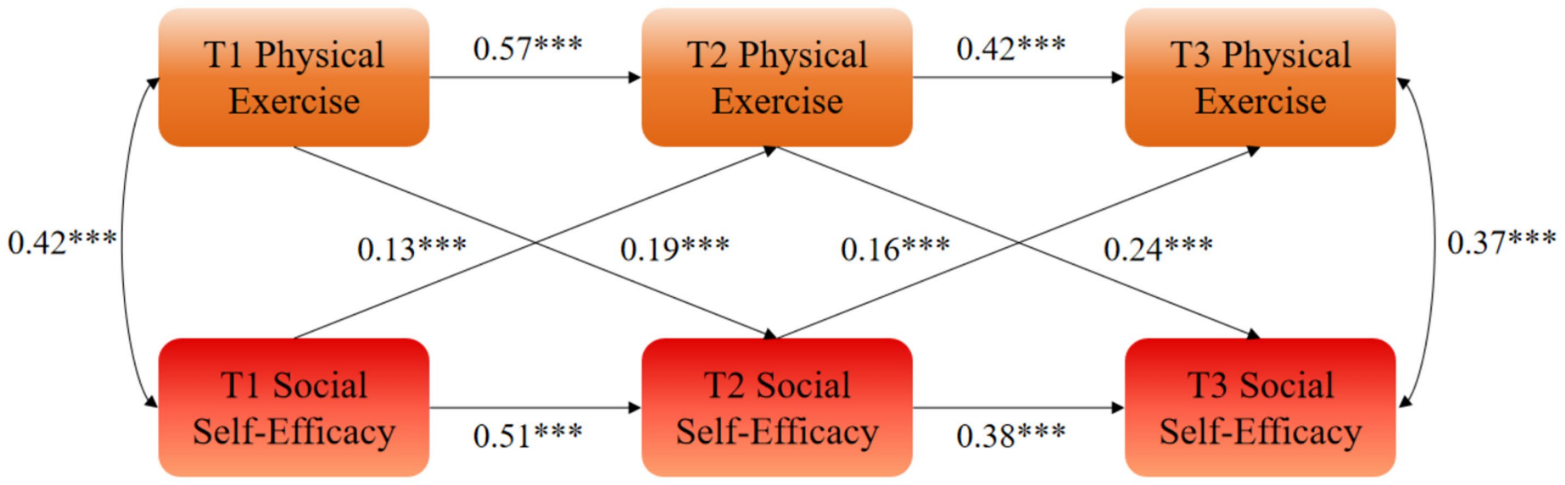
Cross-lagged analysis between physical exercise and social self-efficacy. ***statistical significance at the level of *p* < 0.01.

The cross-lagged effects further revealed that T1 physical exercise significantly and positively predicted T2 social self-efficacy among college students (*β* = 0.19, *p* < 0.01), and T2 physical exercise significantly predicted T3 social self-efficacy (*β* = 0.24, *p* < 0.01). Conversely, T1 social self-efficacy positively predicted T2 physical exercise (*β* = 0.13, *p* < 0.01), and T2 social self-efficacy significantly predicted T3 physical exercise (*β* = 0.16, *p* < 0.01; see Figure 1).

### 3.5 Construction and comparison of competing models

This study employed structural equation modeling (SEM) to examine the longitudinal mediating role of peer acceptance in the bidirectional relationship between physical exercise and social self-efficacy. Four progressive models were constructed for systematic comparison: (1) Baseline Model (M1): Included autoregressive paths for each variable to account for their autocorrelations across different time points, serving as a reference for subsequent models. (2) Forward Path Model (M2): Used T1 physical exercise as the independent variable, T2 peer acceptance as the mediator, and T3 social self-efficacy as the dependent variable, testing the mechanism of T1 physical exercise → T2 peer acceptance → T3 social self-efficacy. (3) Reverse Path Model (M3): Used T1 social self-efficacy as the independent variable, T2 peer acceptance as the mediator, and T3 physical exercise as the dependent variable, examining the pathway T1 social self-efficacy → T2 peer acceptance → T3 physical exercise. (4) Full Path Model (M4): Integrated all the pathways from M2 and M3.

Model fit indices are shown in Table 4. Comparisons between M1 and M2, M3, and M4 indicated significant differences (Δ*χ*^2^ = 152.78, Δdf = 4, *p* < 0.01; Δ*χ*^2^ = 127.45, Δdf = 4, *p* < 0.01; Δ*χ*^2^ = 238.91, Δdf = 8, *p* < 0.01), showing that M2, M3, and M4 fit the data better than M1. Further comparisons between M4 and M2, M3 revealed Δdf₂–₄ = 4, Δdf₃–₄ = 4, Δ*χ*^2^₂–₄ = 86.13, Δ*χ*^2^₃–₄ = 111.46, and p < 0.01, indicating significant differences, with M4 outperforming both M2 and M3. These results suggest that M4 provides the best representation of the relationships among physical exercise, peer acceptance, and social self-efficacy (Table 5).

**Table 4:** Fit indices of competing models.

Model	Model fitting parameters						
	*χ* ^2^	df	CFI	TLI	RMSEA	SRMR	*p*
M1	324.589	24	0.823	0.731	0.136	0.184	<0.01
M2	171.809	20	0.918	0.847	0.105	0.093	<0.01
M3	197.134	20	0.901	0.821	0.113	0.108	<0.01
M4	85.678	16	0.968	0.932	0.076	0.054	<0.01

M, Model; *χ*^2^ = chi-square; df, degrees of freedom; CFI, Comparative Fit Index; TLI, Tucker-Lewis Index; RMSEA, Root Mean Square Error of Approximation; SRMR, Standardized Root Mean Square Residual.

**Table 5:** Longitudinal mediation effects of peer acceptance in the cross-lagged model.

Mediation pathway	*β*	SE	95% CI (Lower, Upper)
T1 Physical Exercise → T2 Peer Acceptance → T3 Social Self-Efficacy	0.071***	0.019	(0.038, 0.108)
T1 Social Self-Efficacy → T2 Peer Acceptance → T3 Physical Exercise	0.053***	0.016	(0.025, 0.084)

***statistical significance at the level of *p* < 0.01.

Path analysis of the optimal model (M4) showed that T1 peer acceptance positively predicted T2 physical exercise (*β* = 0.145, *p* < 0.01) and T2 social self-efficacy (*β* = 0.132, *p* < 0.01). Moreover, T1 physical exercise and T1 social self-efficacy significantly predicted T2 peer acceptance (*β* = 0.213, *p* < 0.01; *β* = 0.186, *p* < 0.01). This predictive pattern was also observed between T2 and T3: T2 peer acceptance significantly predicted T3 physical exercise (*β* = 0.287, *p* < 0.01) and T3 social self-efficacy (*β* = 0.334, *p* < 0.01), while T2 physical exercise and T2 social self-efficacy significantly predicted T3 peer acceptance (*β* = 0.198, *p* < 0.01; *β* = 0.126, *p* < 0.01).

**Figure 2:**
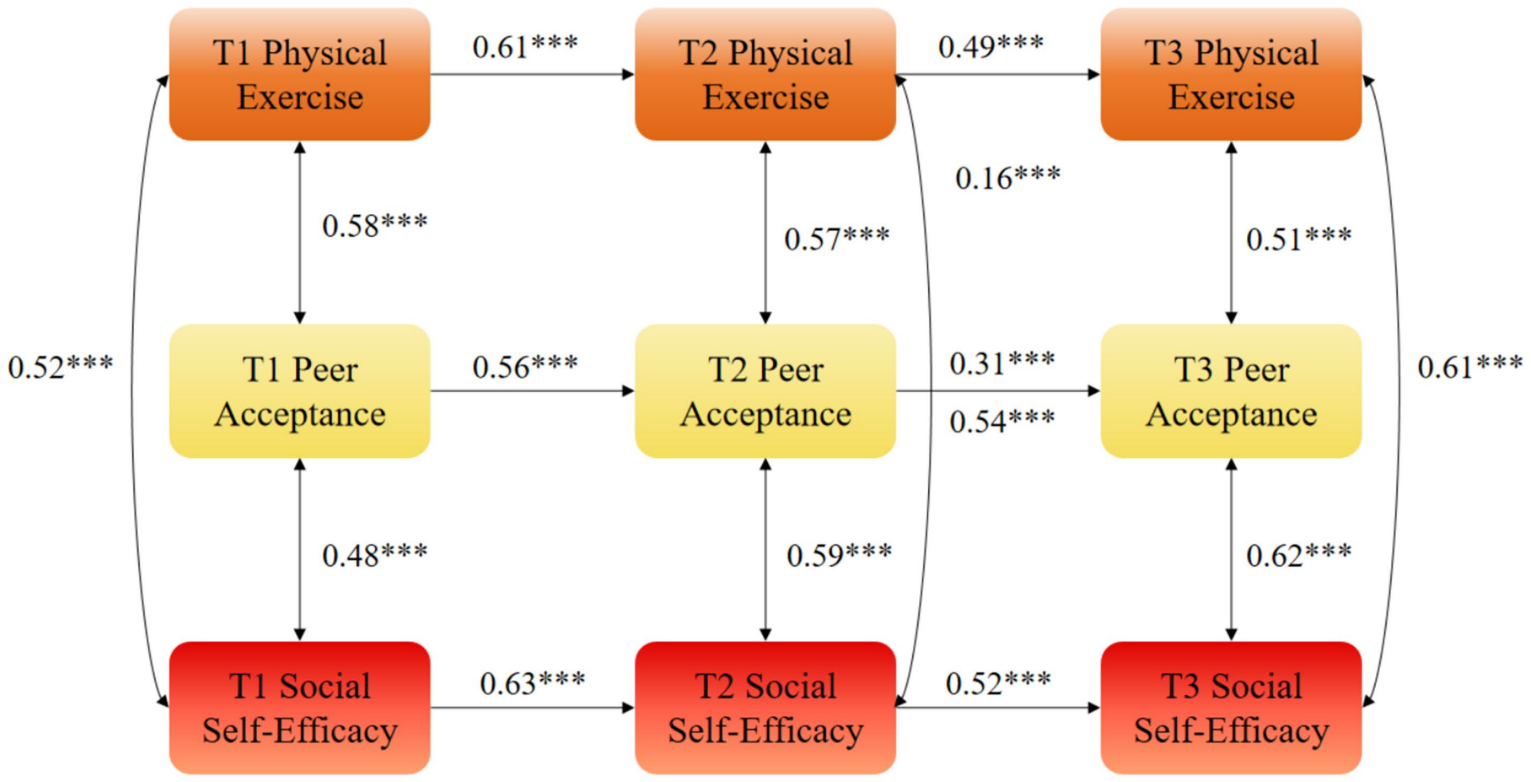
Autoregressive model M1 of physical exercise, peer acceptance, and social self-efficacy. ***statistical significance at the level of *p* < 0.01.

**Figure 3:**
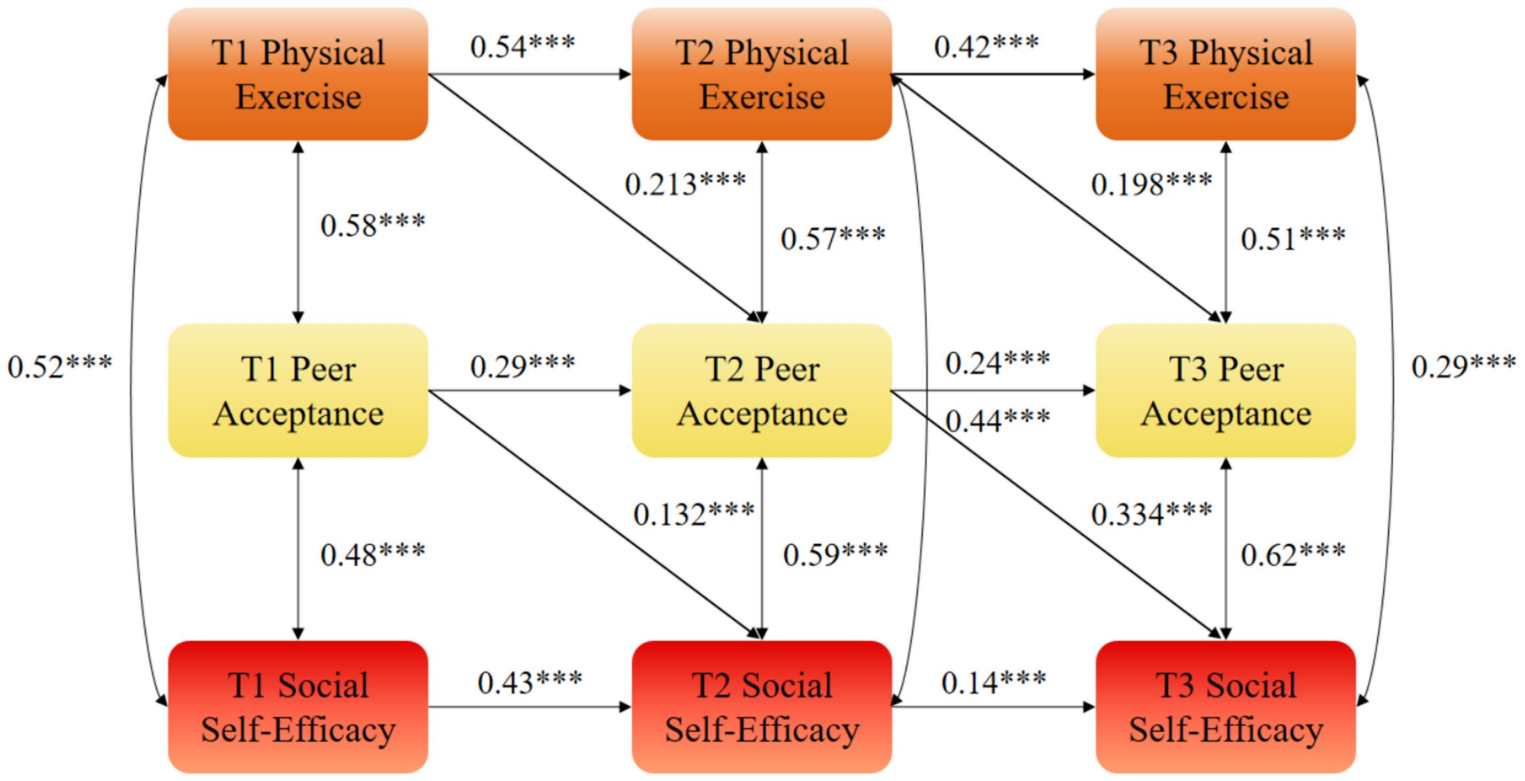
Autoregressive model M2 of physical exercise, peer acceptance, and social self-efficacy. ***statistical significance at the level of *p* < 0.01.

**Figure 4:**
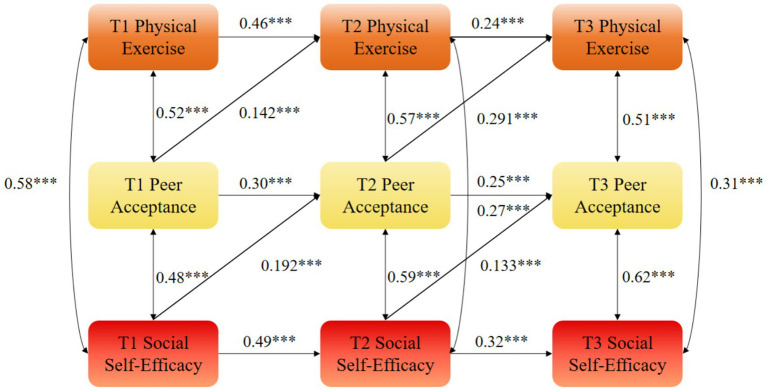
Autoregressive model M3 of physical exercise, peer acceptance, and social self-efficacy. ***statistical significance at the level of *p* < 0.01.

**Figure 5:**
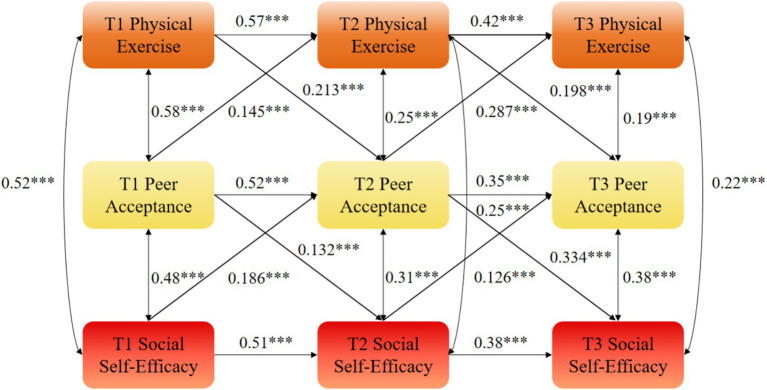
Autoregressive model M4 of physical exercise, peer acceptance, and social self-efficacy. ***statistical significance at the level of *p* < 0.01.

While the mediation effects (*β* = 0.071 and *β* = 0.053) appear numerically modest, they represent approximately 30% of the total cross-lagged effect of physical exercise on social self-efficacy, indicating that peer acceptance plays a substantial role in the mechanism through which sports participation enhances social self-efficacy. From a practical perspective, this demonstrates that fostering supportive peer environments during physical activity is a meaningful way to amplify the psychological benefits of exercise (Figures 2–5).

## 4 Discussion

### 4.1 Longitudinal relationship between physical exercise and social self-efficacy

Using a three-wave longitudinal tracking design, this study revealed a significant bidirectional predictive relationship between physical exercise and social self-efficacy among university students. These results confirm the facilitative effect of physical exercise on social self-efficacy and reveal a reverse relationship in which social self-efficacy promotes engagement in physical exercise, suggesting a mutually reinforcing dynamic cycle between these variables.

Regarding the impact of physical exercise on social self-efficacy, the present findings are consistent with previous research and offer insights into the underlying mechanisms. Based on Social Cognitive Theory, physical exercise, as a highly structured form of social practice, offers individuals abundant opportunities for mastery experiences and social feedback (Beauchamp et al., 2019). During sports participation, university students continually accumulate successful experiences in effectively managing social situations by completing challenging athletic tasks, cooperating with teammates, and resolving team conflicts. Such mastery experiences are among the most powerful sources of information for enhancing social self-efficacy.

Furthermore, physical exercise influences social self-efficacy by altering individuals’ physiological arousal states. Regular physical activity effectively reduces cortisol levels while increasing the secretion of endorphins and dopamine, which in turn improve mood and reduce social anxiety and depressive tendencies (Pressman et al., 2019). When individuals are in a positive and stable emotional state, they are more likely to make favorable evaluations of their own social abilities, thereby strengthening social self-efficacy.

The reverse relationship—whereby social self-efficacy predicts increased physical exercise—has received limited attention in prior research. These findings extend understanding of the reciprocal dynamics between social competence and behavioral engagement. From the Theory of Planned Behavior, behavioral intention is the direct antecedent of behavior and is jointly influenced by attitude, subjective norms, and perceived behavioral control. University students with higher levels of social self-efficacy often possess greater confidence and competence in interpersonal communication, making them more likely to perceive themselves as capable of obtaining social support through physical exercise. As a result, they develop a more positive attitude toward participation. At the same time, individuals with high social self-efficacy are better able to build positive interpersonal relationships within sports teams and obtain greater social recognition and emotional support, and this positive feedback further strengthens their intrinsic motivation to engage in physical exercise.

However, contextual conditions warrant critical attention. In competitive or exclusionary sport environments characterized by high performance pressure, social comparison, and selective acceptance, the beneficial effects may be attenuated or reversed. Students with lower skill levels or athletic abilities may experience repeated failures, negative peer feedback, and social rejection within such contexts, which could undermine rather than enhance their social self-efficacy. Similarly, in team sports environments lacking inclusive leadership or fostering discriminatory practices, individuals from marginalized groups may experience peer rejection rather than acceptance, potentially exacerbating social anxiety and reducing their willingness to participate. These scenarios underscore the importance of not merely promoting sports participation but ensuring that the social environment is genuinely supportive, inclusive, and non-threatening.

### 4.2 Longitudinal mediating role of peer acceptance

One of the most important findings of this study is that peer acceptance longitudinally mediates the bidirectional relationship between physical exercise and social self-efficacy. The validation of this mediating mechanism not only deepens our understanding of the psychological benefits of physical exercise but also provides a key theoretical basis for practical interventions.

From the forward-mediation pathway of “physical exercise → peer acceptance → social self-efficacy,” the mechanism by which physical exercise promotes social self-efficacy through enhancing peer acceptance can be explained from multiple theoretical perspectives. First, according to social exchange theory, physical exercise offers individuals a platform to demonstrate their value and abilities (Yan et al., 2016). In team sports, individuals can convey signals of their social value to peers by demonstrating athletic skills, teamwork spirit, and leadership qualities, thereby gaining greater acceptance from peers. Second, from the perspective of social identity theory, physical exercise fosters a shared group identity and a sense of belonging (Stevens et al., 2017). Individuals participating in the same sport often develop a “we” identity, which facilitates mutual acceptance and support within the group.

A more in-depth mechanism lies in the high-frequency interactions inherent in sports activities, which provide individuals with opportunities to display and develop social skills. Within sports contexts, individuals must engage in effective communication, coordinate actions, regulate emotions, and resolve conflicts—complex social behaviors that, when successfully executed, not only enhance actual social competence but also offer direct evidence that “I can effectively manage social relationships.” When such behaviors receive positive feedback and acceptance from peers, individuals’ beliefs in their social capabilities are reinforced, thereby increasing social self-efficacy.

Conversely, the reverse mediation pathway of “social self-efficacy → peer acceptance → physical exercise” reveals a more complex dynamic process. Individuals with higher social self-efficacy—more confident in their social abilities—tend to behave more naturally, positively, and proactively in interpersonal interactions. Such positive social behaviors are more likely to earn the affection and acceptance of others and to create more opportunities for social connections. When these individuals experience higher levels of peer acceptance in daily life, they are more likely to be invited to participate in various physical activities or to be intrinsically motivated to initiate and engage in exercise to maintain and strengthen these positive social relationships.

It is noteworthy that the mediating role of peer acceptance shows a certain degree of contextual sensitivity. The importance of peer acceptance may vary considerably across different forms of physical exercise. In individual sports such as swimming or running, the role of peer acceptance may be relatively limited, whereas in team sports such as basketball or football, peer acceptance is likely to play a much more critical role. Moreover, personality traits may also moderate this mediating effect. For example, individuals with introverted personalities may be more sensitive to peer acceptance, whereas extroverted individuals may have a stronger capacity to directly improve in social self-efficacy from physical exercise.

### 4.3 Developmental stage specificity and individual differences

This study focuses on university students, whose developmental stage provides an important context for interpreting the findings. The university period is a critical stage for constructing social identity and establishing interpersonal relationship patterns, during which peer relationships are particularly significant. Compared with secondary school, university students face greater social changes and adaptation challenges, requiring them to rebuild interpersonal networks in a new social environment. In this context, physical exercise, as a relatively safe and structured social platform, serves as an important resource for social adaptation.

However, it is important to note that the patterns identified in this study may not be directly generalizable to other age groups. For children and adolescents, peer acceptance may play an even more prominent role, as their self-concept is more strongly dependent on external evaluation. Conversely, for adults, factors such as occupational achievement and family relationships may be more influential in shaping social self-efficacy, and the pathway from physical exercise to social self-efficacy via peer acceptance may be comparatively weaker.

Gender differences also warrant attention. Previous research has shown that women tend to place greater emphasis on emotional support and close connections in interpersonal relationships, whereas men are more focused on status and competition. Such differences may influence the relationship patterns among physical exercise, peer acceptance, and social self-efficacy. For example, among female university students, cooperative and supportive experiences in sports may be more readily translated into a sense of peer acceptance, whereas for male students, experiences of achievement and victory in sports may more directly enhance their social self-efficacy.

### 4.4 Theoretical contributions and innovations

The primary theoretical contribution of this study lies in its rigorous longitudinal design, which validates the dynamic mechanism through which physical exercise influences social self-efficacy, particularly the mediating role of peer acceptance. This finding enriches the application of social cognitive theory in sport psychology and provides a new theoretical perspective for understanding the psychosocial benefits of physical exercise.

Conventional research often treats physical exercise as a singular factor affecting mental health, neglecting the complex social interaction mechanisms involved in this process. By introducing peer acceptance as a key mediating variable, this study reveals the social pathway through which physical exercise benefits mental health, highlighting the critical role of interpersonal relationships in individual psychological development. Such an integrative theoretical framework offers stronger explanatory power and opens new avenues for future research.

More importantly, the bidirectional influence pattern identified in this study challenges the traditional unidirectional causal perspective. Social self-efficacy is not only an outcome of physical exercise but also an antecedent variable that promotes participation in it. This mutually reinforcing dynamic relationship provides new insights into understanding behavior change and maintenance. It suggests that effective intervention strategies should not focus solely on changing a single variable but also on initiating and sustaining such positive feedback loops.

### 4.5 Practical implications and applied value

The findings of this study hold considerable practical significance for university physical education and student mental health promotion. The results not only confirm that physical exercise is an effective means of enhancing social self-efficacy among university students, but also reveal that its impact is primarily achieved by fostering peer acceptance. This suggests that, when designing sports programs, physical educators should go beyond improving athletic skills or physical fitness and intentionally create environments that encourage positive peer interaction and mutual acceptance.

In practice, this can be achieved by integrating more cooperative elements into activities so that participants rely on and support one another to achieve shared goals. A positive team culture that values mutual encouragement, inclusiveness, and collective growth can further strengthen these effects. Providing diverse opportunities for participation allows students of different skill levels to feel valued and a sense of belonging. Regular team-building activities can deepen trust and understanding among team members, while timely and constructive feedback can help address issues that may undermine peer relationships.

For mental health educators, the findings highlight the value of incorporating physical exercise into interventions to improve social self-efficacy. Traditional social skills training often relies on classroom-based instruction or group discussions, which lack the immersive, real-world practice that sports naturally provide. Collaborations between mental health professionals and sports departments could lead to the development of exercise-based psychological interventions that enable students to practice and consolidate social skills in authentic, dynamic contexts.

At the individual level, the results offer guidance for personal growth. Students with lower social self-efficacy may be encouraged to participate in sports as an accessible way to gradually build social confidence by forming positive peer relationships. For those who already possess higher levels of social self-efficacy, engaging in leadership or organizational roles in sports activities can provide opportunities to further refine and strengthen their social competencies.

### 4.6 In-depth analysis of potential mechanisms

Beyond the core mediating role of peer acceptance, the results of this study also suggest other potential pathways worth exploring. From a neuroscience perspective, physical exercise can promote brain plasticity, particularly improving the structure and function of brain regions associated with social cognition, such as the prefrontal cortex and superior temporal sulcus. Enhancements in these regions may directly improve individuals’ ability to process social information, thereby boosting social self-efficacy.

From a physiological perspective, the regulation of neurotransmitter systems through physical exercise may represent an important pathway influencing social self-efficacy. Exercise can increase levels of serotonin and dopamine, neurotransmitters that are closely linked not only to emotional regulation but also to social behavior and cognition. Increased serotonin may enhance individuals’ sociability and cooperative tendencies, while elevated dopamine may strengthen motivation and vitality in social contexts.

From a cognitive perspective, physical exercise may enhance social self-efficacy by improving executive functions, including working memory, cognitive flexibility, and inhibitory control. Strong executive function provides a cognitive foundation for effective social interaction, enabling individuals to better understand social situations, predict others’ behaviors, and regulate their own emotions and actions. As executive function improves, individuals are likely to perform better in social contexts, thereby enhancing their social self-efficacy.

## 5 Conclusions, recommendations, and future directions

### 5.1 Conclusion

Based on a three-wave longitudinal tracking design, this study systematically examined the dynamic relationships among physical exercise, peer acceptance, and social self-efficacy in college students and reached the following main conclusions:

#### 5.1.1 Establishment of a bidirectional facilitative relationship

A significant bidirectional longitudinal predictive relationship exists between physical exercise and social self-efficacy. Physical exercise significantly predicts subsequent levels of social self-efficacy (*β* = 0.19–0.24, *p* < 0.01), while social self-efficacy likewise predicts future engagement in physical exercise (*β* = 0.13–0.16, *p* < 0.01). This mutually reinforcing dynamic offers a new perspective on the psychosocial benefits of physical activity, moving beyond the limitations of a traditional, unidirectional causal framework.

#### 5.1.2 Key mediating role of peer acceptance

Peer acceptance plays a significant longitudinal mediating role in both directions of the relationship between physical exercise and social self-efficacy. Specifically, physical exercise at Time 1 predicts higher social self-efficacy at Time 3 through increased peer acceptance at Time 2 [*β* = 0.071, 95% CI (0.038, 0.108)], while social self-efficacy at Time 1 predicts greater participation in physical exercise at Time 3 through the same mediator [*β* = 0.053, 95% CI (0.025, 0.084)]. This finding highlights the central role of interpersonal relationships in realizing the psychological benefits of physical activity.

#### 5.1.3 Stable longitudinal association pattern

All three variables demonstrated strong stability over the 6-month tracking period, with autoregressive coefficients ranging from 0.38 to 0.57. The cross-lagged effects remained consistent in both direction and statistical significance, indicating a relatively stable developmental trajectory that provides a reliable foundation for the design of long-term intervention strategies.

#### 5.1.4 Measurement invariance over time

All measurement instruments met the strict requirements for longitudinal measurement invariance, including configural, weak, strong, and strict equivalence. This ensures the internal validity and credibility of the study’s findings.

### 5.2 Recommendations

#### 5.2.1 Recommendations for reforming physical education in higher education

University physical education programs should shift from a traditional model that overemphasizes skill instruction and physical training toward a more socially oriented curriculum that prioritizes interpersonal interaction and relationship-building. This could include increasing the proportion of team sports, integrating cooperative elements into individual sports, and offering specialized courses on sport psychology and team building. Physical education instructors should receive targeted training in social and psychological skills to intentionally foster positive peer interactions during instruction. Strategies such as cooperative learning and peer tutoring can be adopted to create more opportunities for mutual support and encouragement among students. A diversified assessment system should be implemented that evaluates not only athletic skills but also teamwork, communication, and social adaptability. Furthermore, a comprehensive social support network for physical activity should be established, including well-structured student sport clubs that cater to different interests and abilities. Regular inter- and intra-university sport exchanges, sport partner-matching systems, and volunteer-based sport programs can help broaden students’ social circles, enhancing their sense of belonging and peer acceptance in sports contexts.

Concrete Implementation Pathway: (1) Phase 1 (Months 1–3): Audit existing PE curriculum and identify 3–5 high-enrollment courses suitable for cooperative redesign. Develop cooperative game modules that require interdependence and mutual support. Train 15–20 PE instructors in cooperative learning and social skills coaching techniques. (2) Phase 2 (Months 4–6): Pilot the redesigned courses with volunteer student cohorts; collect feedback on peer interactions and social comfort. Simultaneously, establish a peer-matching system using student surveys to group individuals by interests, skill level compatibility, and personality fit for team formation. (3) Phase 3 (Months 7–12): Scale successful modules to 50% of PE courses. Launch structured sport clubs with formal peer mentorship roles; develop a recognition system that rewards cooperative behaviors and peer support, not just athletic achievement. Introduce monthly interdepartmental sport exchanges.

#### 5.2.2 Recommendations for integrating mental health education

Mental health education centers and physical education departments should collaborate to develop sport-based psychological intervention programs to improve social self-efficacy. These programs should integrate structured physical activities with psychological training to help students build social confidence. For those with social anxiety or difficulties in social interaction, low-intensity, low-threat sports can be introduced initially, gradually increasing their confidence and competence in social participation. A preventive intervention system should also be established, leveraging the bidirectional relationship identified in this study. For freshmen with low social self-efficacy, encouraging participation in physical activities can help prevent social adaptation problems, while students with low engagement in physical exercise should be monitored for potential deficits in social confidence and provided with tailored psychological support. Integrating physical activity into the overall framework of mental health education can complement traditional counseling and group guidance, with differentiated sport-based approaches designed for various types of psychological challenges.

#### 5.2.3 Recommendations for policy development and resource allocation

Universities should increase investment in sports facilities, particularly those suitable for group activities, and extend opening hours to facilitate student-organized physical activities. A cross-departmental collaboration mechanism involving the physical education department, mental health education center, and student affairs should be established to jointly design and implement comprehensive programs that promote students’ overall development. Regular cross-departmental training and exchanges can improve staff expertise and coordination capacity. Policy support should also be strengthened by increasing the weight of sports participation and social skills in comprehensive student evaluations, thereby encouraging active engagement in physical activity. Incentive mechanisms should be introduced to recognize and reward faculty and staff who demonstrate outstanding contributions to promoting student participation in sports and social development.

Operational Specifications: Establish a joint PE-Mental Health Task Force that meets biweekly. Create standardized screening protocols to identify first-year students with low social self-efficacy for targeted low-intensity sport recommendations. Design 8-week sequential modules: weeks 1–2 individual activities (yoga, tai chi); weeks 3–4 partner-based activities; weeks 5–8 small group team sports. Assign trained peer mentors to facilitate transition and provide encouragement. Conduct mid-program and post-program assessments of social anxiety, peer connection, and intention to continue sports.

#### 5.2.4 Recommendations for personal development guidance

For students with lower social self-efficacy, non-competitive activities such as yoga, tai chi, or brisk walking are recommended as starting points, allowing them to gradually build confidence in a relaxed environment. Those with higher social self-efficacy can be encouraged to take on more organizational and leadership roles within sports teams to further develop their social leadership skills. Students should be guided to create personalized physical activity and social development plans based on their individual characteristics, viewing sport participation as an integral part of personal growth. It is also important to recognize the long-term cumulative effects of physical activity on social competence and to maintain regular participation over time.

Specific Departmental Responsibilities: (1) Physical Education Department: Expand court and gym hours to 6 a.m.–10 p.m.; reserve 20% of court time for student-organized clubs; allocate 30% of equipment budget to cooperative sport materials. (2) Mental Health Center: Integrate sport participation into social anxiety intervention protocols; provide quarterly training to PE staff on recognizing and supporting students with social difficulties; co-develop sport-based psychological assessment tools. (3) Student Affairs: Revise the student evaluation rubric to allocate 10% weight to ‘social engagement and peer relationships’; establish a sports participation incentive system; mandate cross-departmental collaboration goals for all departments involved.

### 5.3 Limitations and directions for future research

#### 5.3.1 Main limitations of this study

First, regarding sample representativeness, this study was conducted at a single university (Zhengzhou University), limiting the geographical and cultural representativeness of the sample. Students from different regions and types of institutions (e.g., elite “985/211” universities versus ordinary undergraduate institutions, comprehensive universities versus specialized colleges) may differ significantly in their patterns of sports participation, peer relationship characteristics, and levels of social self-efficacy. Furthermore, the participants in this study were predominantly Han Chinese, so the applicability of the findings to ethnic minority student populations remains to be verified.

Second, the tracking period was relatively short at 6 months, which may have been insufficient to capture the long-term effects of physical exercise on social self-efficacy. As a lifestyle behavior that requires sustained engagement, the psychosocial benefits of physical activity may take longer to manifest fully. Short-term tracking also makes it difficult to observe potential nonlinear developmental trajectories or critical turning points.

Third, there were limitations in measurement methods. Although the self-report scales used in this study possess sound psychometric properties, reliance on self-reports may still be subject to biases such as social desirability and common method variance. In particular, for measuring physical activity levels, objective behavioral indicators (e.g., data from wearable activity monitors) may be more accurate than self-reports.

Fourth, the selection of variables was not exhaustive. While this study focused on the mediating role of peer acceptance, the mechanisms linking physical exercise and social self-efficacy are likely more complex, involving multiple mediating and moderating variables. Factors such as individual athletic competence, coaching style, and the characteristics of specific sports disciplines may also influence this relationship.

Fifth, individual differences were insufficiently considered. Although basic demographic variables were controlled for, differences in personality traits, baseline athletic skills, and prior sports experience were not adequately addressed. Such individual differences may moderate the relationships among physical exercise, peer acceptance, and social self-efficacy.

Sixth, Individual Heterogeneity in Change Trajectories: The fixed 3-month measurement interval may not be uniformly optimal for all variables or all participants. Different individuals display heterogeneous patterns of change at varying rates—some may show rapid improvements in social self-efficacy following increased sports participation, while others require longer periods to consolidate peer relationships and psychological gains.

#### 5.3.2 Directions for future research

First, future research should expand the sample scope and extend the tracking period. Multi-center, multi-region research designs that include different types of institutions and geographical areas are recommended to improve the external validity of the findings. The tracking period should be extended to one or 2 years, with more frequent measurement points to better capture the developmental trajectories of the variables. A cohort design beginning at student enrollment could be adopted to investigate how critical transition periods moderate these relationships.

Second, multiple measurement methods should be integrated. Objective behavioral measures, physiological indicators, and subjective reports should be combined to establish a more comprehensive assessment framework. For physical activity, wearable devices can be used to monitor actual exercise volume and patterns; for peer acceptance, peer nomination methods and social network analysis can be employed to assess an individual’s actual position within a group; for social self-efficacy, task-based behavioral assessments in specific social scenarios can be used, thus creating a multi-source validated composite index.

Third, future studies should explore more thoroughly the mechanisms and moderating factors underlying the effects of physical exercise on social self-efficacy. At the physiological level, neuroimaging techniques can be applied to examine structural and functional changes in the brain, and biochemical markers can help reveal neurobiological pathways. At the psychological level, mediators such as emotion regulation and cognitive flexibility, as well as moderators such as achievement goal orientation and self-determined motivation, should be examined. At the social level, factors such as social support, team cohesion, and group identity merit consideration. Attention should also be paid to the moderating effects of individual differences and situational factors.

Fourth, targeted intervention programs should be developed, and research on the effects of different types of sports should be refined. Intervention programs based on the study’s findings should focus on creating positive peer interaction environments, incorporating cooperative games, and establishing peer support systems, with their effectiveness tested through randomized controlled trials. It is also important to distinguish the differential effects of various types of physical activity—for example, comparing individual versus team sports and aerobic versus anaerobic exercise—on peer acceptance and social self-efficacy. The influence of exercise parameters such as intensity, frequency, and duration should be explored to provide evidence-based guidance for personalized exercise prescriptions.

Additionally, future research should systematically examine whether the type of physical activity serves as a moderator of the relationships among physical exercise, peer acceptance, and social self-efficacy, thereby providing more nuanced guidance for tailoring interventions to different student populations.

## Data Availability

The raw data supporting the conclusions of this article will be made available by the authors, without undue reservation.
